# Effects of Sesame Consumption on Inflammatory Biomarkers in Humans: A Systematic Review and Meta-Analysis of Randomized Controlled Trials

**DOI:** 10.1155/2021/6622981

**Published:** 2021-11-01

**Authors:** Shabnam Rafiee, Roghaye Faryabi, Alireza Yargholi, Mohammad Ali Zareian, Jessie Hawkins, Nitin Shivappa, Laila Shirbeigi

**Affiliations:** ^1^Department of Traditional Medicine, School of Persian Medicine, Tehran University of Medical Sciences (TUMS), Tehran, Iran; ^2^Integrative Health, Franklin School of Integrative Health Sciences, Franklin, TN, USA; ^3^Cancer Prevention and Control Program, University of South Carolina, Columbia, SC 29208, USA; ^4^Department of Epidemiology and Biostatistics, Arnold School of Public Health, University of South Carolina, Columbia, SC 29208, USA

## Abstract

**Objectives:**

Existing evidence produces conflicting findings regarding the effect of sesame intake on inflammatory biomarkers; this knowledge gap has yet to be met through systematic review and meta-analysis. This meta-analysis of randomized, controlled clinical trials (RCTs) was conducted to evaluate the effects of sesame consumption on markers of inflammation in humans.

**Methods:**

PubMed, Scopus, and the Cochrane Database of Systematic Reviews were searched through August 2020 to identify relevant papers for inclusion. Using the random-effects model, data were evaluated as weighted mean differences (WMD) with 95% confidence intervals (CI). Cochrane's *Q* and *I*-squared (*I*^2^) tests were used to identify within-studies heterogeneity.

**Results:**

Seven RCTs with 310 participants (157 intervention and 153 control) were included in the meta-analysis. Sesame consumption reduced serum level interleukin-6 (IL-6) (WMD − 0.90; 95% CI (−1.71, −0.09), *I*^2^ = 80.4%) compared to the control group. However, sesame intake had no significant effects on C-reactive protein (CRP) and tumor necrosis factor-*α* (TNF-*α*) compared to the control group. Subgroup analysis identified a reduction in serum CRP, TNF-*α*, and IL-6 concentration among studies with participants who had a higher level of these biomarkers at baseline, those which used sesamin capsules, and those with a bigger sample size, those conducted in Asia, and studies on females.

**Conclusion:**

Sesame consumption reduced serum levels of IL-6 but did not affect CRP and TNF-*α* in humans. Additional trials should be conducted utilizing a larger and longer treatment duration, along with studies using different sesame formulations (capsule, oil, and seed) and conducting on participants with varied health conditions.

## 1. Introduction

Low-grade chronic inflammation, characterized by the chronically low levels of inflammatory biomarkers, is associated with type 2 diabetes, cardiovascular disease, aging, obesity, and different kinds of cancer [1–3]. Interleukin-6 (IL-6), tumor necrosis factor-*α* (TNF-*α*), and C-reactive protein (CRP) are among the most commonly studied inflammatory biomarkers [4]. IL-6, with both anti-inflammatory and pro-inflammatory properties, is produced by macrophages and T-cells [5, 6]. TNF-*α* is a pro-inflammatory cytokine produced by macrophages following inflammatory stimuli [7]. CRP, an acute-phase protein, is secreted from the liver [8] as a response to elevated concentrations of IL-6 and TNF-*α* [9]. Given the role of inflammation in the pathogenesis of chronic illness, anti-inflammatory agents such as exercise, antioxidants, and nonsteroidal drugs are practical preventive measures for chronic diseases [10, 11]. Dietary factors are also considered the main alternative treatments for low-grade inflammation [12, 13]. Among dietary factors, nutraceuticals and plant oils have garnered great attention due to the adverse effects and high cost of many medications [14, 15]. Several studies indicated anti-inflammatory effects of oil crop intake including flaxseed, olive, and canola oils both in Asian [16, 17] and Western countries [18, 19].

Sesame (*Sesamum indicum*) is a culinary ingredient that has been used in food preparation for thousands of years, especially in Asian countries [20]. The seeds contain an abundance of polyunsaturated fatty acids (PUFA), vitamin E, phytosterol, fiber, and bioactive lignans (*sesamin*, *sesamol*, *sesamolin*, and *episesamin*), which produce antioxidant and anti-inflammatory activity [21–23]. One of the most abundant lignans in sesame (*sesamin*) has been found to exhibit beneficial effects on inflammatory markers [24], hyperglycemia [25], hypertension [26], and hyperlipidemia [27] in humans. Sesamin is found in many sesame-containing products, including sesame oil, sesame seeds, sesame meals, and sesame-based supplements [28].

Evidence from animal studies reveals that sesamin improves hyperglycemia, reduces inflammation, and improves insulin resistance in diabetic mice [29]. However, human studies have produced conflicting results [24, 30–35]. While some studies found that sesame consumption reduces inflammatory biomarkers levels [33, 35], others did not find the same benefits [24, 34]. This study is the first systematic review and meta-analysis of randomized controlled trials, with the purpose of quantifying the overall effect of sesame consumption on inflammatory biomarkers in humans.

## 2. Materials and Methods

This systematic review and meta-analysis utilize the Preferred Reporting Items for Systematic Reviews and Meta-Analyses (PRISMA) guidelines [36]. The protocol was registered at the Center for Open Science Framework (OSF) database (https://www.osf.io, DOI: 10.17605/OSF.IO/TJ4N8).

### 2.1. Search Strategy

The search included online databases of PubMed, Scopus, and the Cochrane Database of Systematic Reviews continued until August 2020. It was conducted using medical subject headings (MESH) and relevant keywords of the following terms: (“Sesame^*∗*^” OR “Sesame Oil” OR “Sesame Seed” OR “*Sesamum*” OR “*Sesamin* supplement”) AND (“adiponectin” OR “leptin” OR “TGF-*β*” OR “high sensitive CRP” OR “IL-10” OR “IL-8” OR “TNF” OR “IL-6” OR “hs-CRP” OR “CRP” OR “Biological marker” OR “Systemic inflammation” OR “Interleukin” OR “Interleukin-1*β*” OR “adipokines” OR “myokine” OR “Neurogenic Inflammation” OR “Inflammation Mediator” OR “Monocyte chemotactic protein 1” OR “Intercellular adhesion molecule-1” OR “p-selectin” OR” eselectin” OR “Matrix metalloproteinase” OR “Acute phase reactant” OR “Cytokine” OR “Transforming growth factor beta” OR “C- Reactive protein” OR “Tumor necrosis factor” OR “Interleukin-6” OR “Interleukin-8” OR “Interleukin-10” OR “Inflammatory biomarker” OR “inflame”^*∗*^). Database search restrictions were not utilized. The Google Scholar, reference lists of the eligible RCTs, and related review articles were also assessed to identify RCTs that were not found in the online database search.

### 2.2. Eligibility Criteria

Randomized controlled clinical trials were included if they met the following criteria: (1) studies which evaluated the effects of consuming sesame or its products (sesame seed, sesame oil, or *sesamin* supplement) on serum concentrations of inflammatory biomarkers, including CRP, TNF-*α*, and IL-6; (2) studies which reported mean or median, standard deviation (SD), and 95% confidence interval on inflammatory biomarkers. Both parallel and crossover designs were included. It was excluded if a study did not evaluate serum inflammatory markers or included sesame in a combination of active ingredients. Two separate investigators (SR and AY) evaluated each article's title and abstract based on the inclusion/exclusion criteria. Discrepancies in data extraction were resolved by the principal investigator (LS).

### 2.3. Data Extraction

Data were extracted independently by two researchers (MAZ and RF) and included the following information: first author's surname, publication year, study location, participants health status, age (mean/range) and sex, number of participants, study design, duration of intervention, sesame product and dosage, characteristics of the placebo/control group, mean and standard deviation (SD) both at baseline and post-intervention. Data from studies that did not report inflammatory measures as SI units were converted to these measures.

### 2.4. Risk of Bias Assessment

The potential risk of bias for each included study was assessed using the Cochrane Collaboration's tool [37]. This tool evaluates factors such as blinding, loss of follow-up, random sequence generation, selective reporting of outcomes, and other potential sources of bias. Studies were ranked as having a high, unclear, or low risk of bias based on those criteria.

### 2.5. Statistical Analysis

Effect sizes, which were calculated from the mean and standard deviations of the change in inflammatory markers, were pooled using a random-effects model and reported as weighted mean difference (WMD) and 95% confidence interval. Mean changes were calculated if the study did not provide those data. The formula used for calculating mean changes was as follows: final mean value minus baseline mean value. Standard deviations were calculated as SD_Change_ = $\sqrt{{\text{SD}}_{\text{Baseline}}^{2}+{\text{SD}}_{\text{Final}}^{2}-\left(2\times r\times {\text{SD}}_{\text{Baseline}}\times {\text{SD}}_{\text{Final}}\right)}$ [38]. A correlation coefficient equal to 0.8 was considered as *R*-value when calculating SD change [38]. Data that was reported as median and interquartile ranges was converted to means and standard deviations with the method described by Hozo et al. [39]. If required, confidence intervals were converted to standard deviations using the formula $\text{SD}=\sqrt{n}\times \text{upper limit}\left(\text{UL}\right)-\text{lower limit}\left(\text{LL}\right)/3.92$ [38]. To identify heterogeneity, we calculated *I*^2^, and values above 50% were interpreted as evidence of heterogeneity. We also conducted subgroup analysis using predefined factors. These included the type of sesame product (i.e., seed, oil, and supplement), baseline serum values, study size, duration, geographic region, sex, and baseline BMI. Sensitivity analyses were conducted to assess the role of each study in the final effect size. Egger's test and visual inspection of funnel plots were used to identify the potential for publication bias [40]. All statistical procedures were conducted using STATA (Version 12.0, Stata Corp, College Station, TX, USA), and statistical significance was defined as *P* < 0.05.

## 3. Results

### 3.1. Study Selection

**Figure 1:**
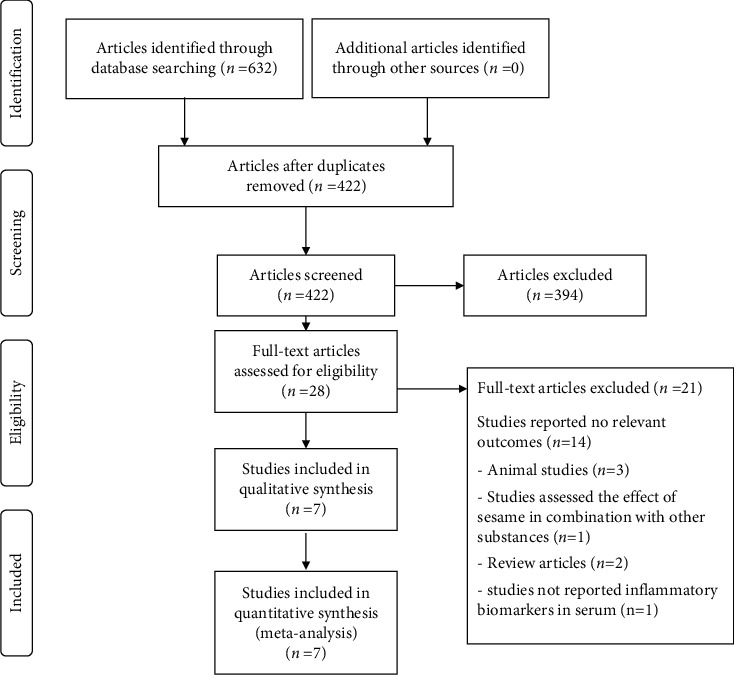
Study selection diagram.

The online database search produced 632 relevant manuscripts, and no additional documents were identified through the evaluation of reference lists. Removal of 210 duplicate manuscripts left 422 abstracts to screen. After screening, 28 studies remained for full-text evaluation. This process eliminated an additional 21 studies: irrelevant outcomes (*n* = 14); review articles (*n* = 2); animal research (*n* = 3); a combination of sesame with other substances (*n* = 1); and failure to evaluate serum biomarkers (*n* = 1). A total of seven studies remained for this analysis (Figure 1).

### 3.2. Study Characteristics

All the identified studies were RCTs published between 2009 and 2019 (see Table 1). The studies reflected 157 participants in treatment groups and 153 participants in control groups and had interventions ranging from 4 weeks to 8 weeks. The 310 participants were aged 16 to 70 years. Two of the seven trials only included males [30, 34], one only included females [33], and the other four trials included both sexes [24, 31, 32, 35]. Four studies were conducted in Iran [31, 33, 35], one in Brazil [30], one in Greece [34], and one study in Australia [24]. Different sesame products were used for the intervention: sesame oil was used in two studies [31, 34], three studies used sesame seeds [24, 30, 32], and two studies used *sesamin*-based supplement [33, 35]. Placebo materials also varied; options included starch capsules [33, 35], sunflower oil [31], a combination of honey, maltodextrin, milk, and caramel coloring [30], and glucosamine [32]. One study did not identify which placebo materials were used [24], and another study did not use the standard of care (regular diet) rather than a placebo [34]. None of the studies reported any significant side effects. Patient's health among the studies varied, one study used patients with type 2 diabetes [35], one used patients with hypertension [34], one used patients with metabolic syndrome [31], one used overweight patients [24], one used semiprofessional athletes [30], one used patients with rheumatoid arthritis [33], and one used patients with knee osteoarthritis [32]. Six of the trials used a parallel design [30–35], and the other used a crossover design [24].

### 3.3. Assessment of the Risk of Bias

After assessing the quality of studies using the six domains of the Cochrane Collaboration's tool, five studies were found to be of high quality, while the others were classified as fair. All studies described which method was used for randomization. Two studies had poor allocation concealment, and five studies were single- or double-blinding (Table 2).

### 3.4. Meta-Analysis

#### 3.4.1. Findings on the Effect of Sesame on CRP

**Figure 2:**
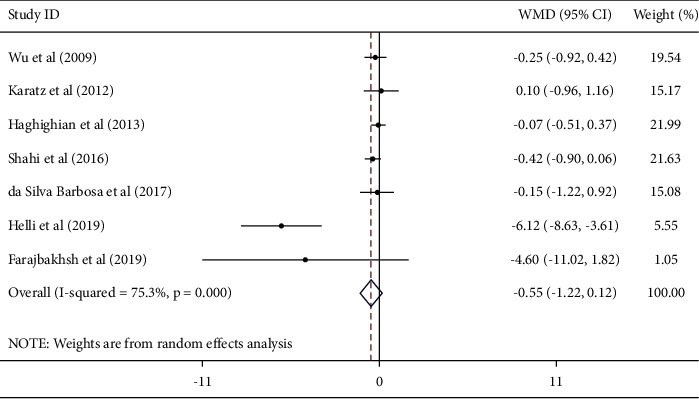
Forest plot showing the effects of sesame consumption on circulating CRP (WMDs and 95% CIs) in humans using the random-effects model. CI: confidence interval; CRP: C-reactive protein; and WMD: weighted mean difference.

Combining the effect sizes from 7 studies, we did not find a significant reduction in serum CRP concentrations after sesame consumption, as compared to control group [weighted mean difference [Weighted Mean Difference (WMD) − 0.55; 95% CI (−1.22, 0.12), *I*^2^ = 75.3%] (Figure 2). The subgroup analysis found that baseline serum CRP levels, baseline BMI, intervention type, duration of treatment, geographic region, sample size, and sex are all sources of heterogeneity. Subgroup analysis also found that CRP was significantly reduced in studies which were conducted in Iran [WMD – 1.38; 95% CI (−2.70, −0.06), *P*=0.039], studies in which participants had higher baseline serum CRP (≥10 mg/L) [WMD – 5.91; 95% CI (−8.25, −3.58), *P* < 0.01], studies conducted on females [WMD – 6.12; 95% CI (−8.63, −3.61), *P* < 0.001], and studies with a larger sample size (≥40) [WMD – 1.38; 95% CI (−2.70, −0.06), *P*=0.039] (Table 3).

#### 3.4.2. Effect of Sesame on IL-6

**Figure 3:**
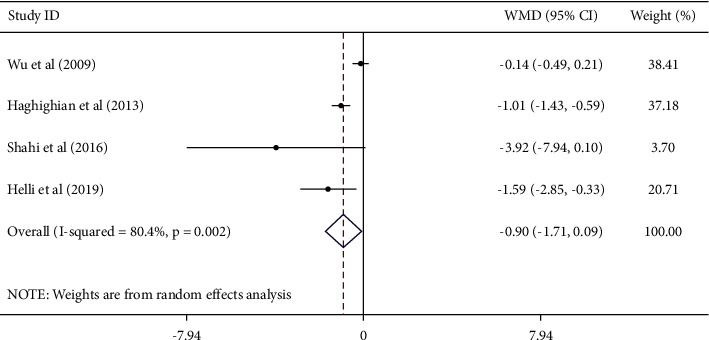
Forest plot showing the effects of garlic supplementation on circulating IL-6 (WMDs and 95% CIs) in adults using the random-effects model. CI: confidence interval; IL-6: interleukin-6; and WMD: weighted mean difference.

Based on the findings of 4 studies, a significant reduction in serum IL-6 was found after sesame consumption [WMD − 0.90; 95% CI (−1.71, −0.09), *I*^2^ = 80.4%] (Figure 3). According to subgroup analyses, intervention type, baseline serum values of IL-6, geographic region, baseline BMI, duration of treatment, and sample size are all sources of heterogeneity. Subgroup analysis also found that sesame consumption decreased serum IL-6 in studies which used *sesamin* capsule and in studies of participants with higher baseline serum IL-6 (≥2 ng/L) [WMD – 1.94; 95% CI (−3.57, −0.30), *P*=0.020], studies conducted in Iran, studies with larger sample sizes (≥40) [WMD – 1.23; 95% CI (−1.95, −0.52), *P* < 0.001], and studies conducted on females [WMD – 1.59; 95% CI (−2.84, −0.33), *P*=0.013] (Table 3).

#### 3.4.3. Effect of Sesame on TNF-*α*

**Figure 4:**
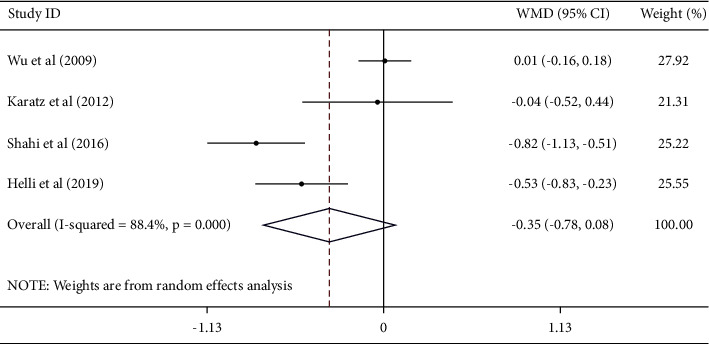
Forest plot showing the effects of sesame consumption on circulating TNF-*α* (WMDs and 95% CIs) in humans using the random-effects model. CI: confidence interval; TNF-*α*: tumor necrosis factor-*α*; and WMD: weighted mean difference.

Pooling the effect sizes from 4 studies, we failed to find a significant reduction in serum TNF-*α* concentrations after sesame consumption [WMD − 0.35; 95% CI (−0.78, 0.08), *I*^2^ = 88.4%] (Figure 4). Subgroup analysis identified intervention type, geographic region, and sample size as the sources of heterogeneity. Subgroup analysis also found that sesame consumption had a significant effect on TNF-*α* in studies which were conducted in Iran, used *sesamin* supplementation, had larger samples (≥40) [WMD − 0.67; 95% CI (−0.95, −0.38), *P* < 0.001], and studies conducted on females [(WMD) – 0.53; 95% CI (−0.82, −0.23), *P*=0.001] (Table 3).

### 3.5. Sensitivity Analysis

A sensitivity analysis was conducted to identify the size of the influence of each study on the overall effect size produced. This analysis found that none of the studies significantly affected the outcome (Supplementary Figures 1–3).

### 3.6. Publication Bias

Publication bias was assessed using visual inspection of funnel plots and the Egger test. Inspection of the funnel plots did not provide any evidence of publication bias (Supplementary Figures 4–6). These observations were confirmed by Egger's linear regression for CRP (*P*=0.12), IL-6 (*P*=0.31), and TNF-*α* (*P*=0.44).

## 4. Discussion

The present meta‐analysis showed that sesame and the consumption of its products could lower serum IL‐6 levels but did not affect serum concentrations of CRP and TNF-*α* in humans. Besides, a significant reduction was identified in serum CRP, TNF-*α*, and IL-6 concentration among studies with participants who had a higher level of these biomarkers at baseline, used *sesamin* capsules, had a larger sample size, were conducted in Asia, and studies with female participants. This is the first systematic review and meta‐analysis to identify the effects of sesame supplementation on serum inflammatory biomarkers.

We found that serum IL-6 decreased followed by sesame consumption. In line with our result, Hsu et al. [41] revealed that administration of different dosages of sesame oil (0, 1, 2, or 4 mL/kg, orally) decreased IL-6 and TNF-*α* in the rat. Another study in microglia cells showed neuroprotective influences of sesamin supplementation by its effects on lowering mRNA levels of IL-6 [42]. Chaval and Forse indicated that mice fed the diet that contained 1% sesamol had lower serum levels of IL-6 and PGES2 in comparison to the control mice [43]. One randomized double-blind, placebo-controlled showed daily consumption of a low-fat muffin plus flaxseed lignan complex (500 mg/d of secoisolariciresinol diglucoside) for 6 weeks did not decrease IL-6 compared to a low-fat muffin in 22 healthy postmenopausal women [44]. The level of lignans in sesame is even higher than in flaxseed, which was previously considered the richest source of lignans [45]. One animal study indicated more beneficial effects of the sesame seed lignan in lowering breast tumor growth compared to flaxseed lignan [46].

We did not find significant effects of total sesame consumption on serum CRP and TNF-*α* concentrations. In consistency with our results, an in vitro study showed supplementation for 12 weeks with 18 g/d of sesame oil did not have a significant effect on TNF-*α* and PGE-2 in cultured blood of healthy male volunteers [47]. In addition, supplementation with 25 g/day sesame seeds for 5 weeks had no beneficial effects on CRP, TNF-*α*, and IL-6 in overweight or obese men and women [24]. In contrast to our findings, one RCT study indicated that 0.5 mL/kg/day sesame oil consumption along with interferon beta-1a decreased TNF-*α* measured in supernatants of peripheral blood mononuclear cells in patients with multiple sclerosis compared to patients who only received interferon beta-1a [48]. In one randomized clinical trial study, supplementation with 360 mg/d flaxseed-derived lignin for twelve weeks decreased C-reactive protein in 39 diabetic women [49]. In an animal study conducted by Chiang et al., sesamin significantly reduced the serum CRP, TNF-*α*, and IL-6 levels in the rat with liver injury received 10 mg/kg sesamin orally [50]. These inconsistent findings might be partially due to different sources of sesame, geographic region, sex, and study sample size.

Using sesame as sesamin capsule decreases CRP, IL-6, and TNF-*α* significantly. This may be due to the high bioavailability of sesame lignan in sesamin capsules compared to sesame seed or sesame oil. In addition, our study also showed that sesame intake lowered CRP, IL-6, and TNF-*α* in studies in which participants had higher baseline serum levels of these markers. Rheumatoid arthritis, knee osteoarthritis, lupus erythematosus, and multiple sclerosis are chronic inflammatory systemic diseases (CIDs) with high serum inflammatory biomarkers [51].

The participants of most included studies had normal serum levels of inflammatory markers at the baseline. Therefore, this may be a reason for observing no significant effects in the meta-analysis. Besides, this study also indicated that sesame intake lowers CRP and IL-6 among participants with high baseline serum levels of these markers. Because nearly all participants had normal serum TNF-*α*, we could not perform subgroup analysis for this biomarker. Although obese individuals have higher concentrations of inflammatory biomarkers than normal weight subjects, this study did not show any anti-inflammatory effects of sesame in both obese (BMI ≥ 30 Kg/m^2^) and non-obese (BMI < 30 Kg/m^2^) persons due to a limited number of studies in each subgroup. Furthermore, the duration of eligible studies (≤8 weeks) might not be long enough to see the possible effects on inflammatory markers. Moreover, sesame is a commonly used oil, especially in Asian countries [20]. The studies conducted in Iran show that sesame consumption declined inflammatory biomarkers. Another reason for failing to find a significant association may be a low number of participants.

**Figure 5:**
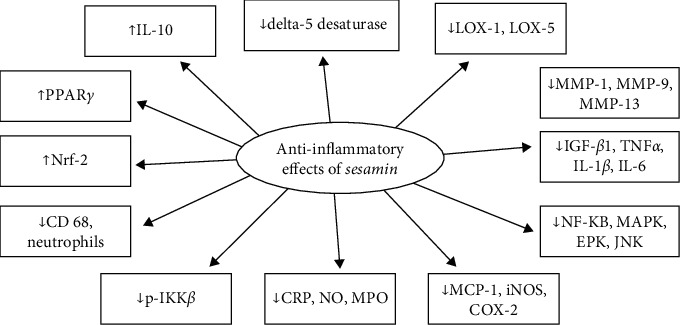
The mechanism of sesame reducing inflammation.

Several mechanisms of action have been identified as responsible for the anti-inflammatory effects. Sesamol (3–100 *μ*M) was found to lessen the production of nitric oxide (NO) and pro-inflammatory cytokines, suppress the expression of inducible nitric oxide synthase (iNOS) and cyclooxygenase-2 (COX-2), and promote Nrf2, an antioxidant pathway. It was also found to block the mitogen-activated protein kinase (MAPK) and NF-*κ*B pathways and decrease reactive oxygen species production, which decreased the inflammatory response [52]. In a study by Chu and colleagues, one subcutaneous injection of sesamol (10 mg/kg, s.c.) was found to significantly reduce the levels of NF-*κ*B, iNOS, and interleukin 1*β* (IL-1*β*) [53]. Another study revealed that sesamol (5–20 *μ*M) significantly inhibits the expression of several matrix metalloproteinases (MMPs), TNF*α*- and IL-1*β*-induced gelatinolysis of MMP-9, MMP-1, and MMP-13 expression, IL-1*β*- and phosphorylation of ERK1/2 or p38 MAPKs in SW1353 cells, a human chondrosarcoma cell line [54]. The same study revealed that oral administration of sesamol (30 mg/kg, p.o.) for 2 weeks attenuated MMP-1 and MMP-9 expression in the cartilage of monosodium iodoacetate (MIA)-induced osteoarthritis in male Wistar rats [54]. Sesamol (0.1–1.0 mg/kg, p.o.) also inhibited the activation of mucosal NF-*κ*B and attenuated the recruitment of inflammatory cells, including CD68 + Kupffer cells and neutrophils [55]. Noteworthy, Chang and colleagues demonstrated that sesamol (2.5–25 *μ*M) treatment in platelets significantly decreased collagen-induced phosphorylation of I*κ*B kinase *β* (IKK*β*) [56] (Figure 5).

This study has several limitations. The total number of clinical trials that qualified for this analysis was small, and more studies are required to deepen our understanding. Additionally, this analysis includes studies conducted on a wide range of dosing protocols, participant ages, underlying health status, and duration. We tried to detect the source of heterogeneity by subgroup analysis. However, because studies were performed in participants with a different health condition and limited number of studies for each subgroup, we could not perform subgroup analysis according to health status. The overall result may have been influenced by the differences in dosing and sourcing of sesame. Subgroup analysis to identify dose-dependence or contrast groups by dosing was not feasible due to variations in the sesame preparations and the difficulty of obtaining accurate dose conversions. In addition, most of the trials included in this analysis were conducted in Asian countries and were small-scale studies assessing effects elsewhere. Finally, the high risk of random allocation and blinding biases found in some studies resulted in lower scientific evidence.

## 5. Conclusion

In conclusion, the consumption of sesame can significantly reduce serum IL-6 levels without any effects on CRP and IL-6. The small numbers of related RCTs and high amounts of heterogeneity among these studies limit generalizability. We recommend that additional high-quality RCTs be conducted on participants with different health conditions, varying the duration of intervention, and multiple forms and dosages of sesame consumption to confirm our findings.

## Figures and Tables

**Table 1:** Characteristics of randomized trials on the effects of sesame consumption on inflammatory biomarkers in humans included in the meta-analysis.

Reference	Location	Subjects and gender	Age, *y*^1^	Design	Intervention type		Duration (wk)	Outcomes	Outcome^2^		Notes about subjects
					Intervention	Control (name and composition)			Intervention	Control	
Helli et al. [33]	Iran	F : 44	S + C:	Parallel	Sesamin capsule (200 mg/d)	Placebo capsules (starch)	6	hs-CRP	hs-CRP (mg/L) : before: 22.28 ± 5.85; after: 17.33 ± 4.05	hs-CRP (mg/L) : before: 20.74 ± 7.43; after: 21.91 ± 7.81	44 patients with rheumatoid arthritis
		S : 22	55.49 ± 5.98					TNF-*α*	TNF (ng/L) : before: 2.17 ± 0.71; after: 1.67 ± 0.58	TNF (ng/L) : before: 2.03 ± 0.82; after: 2.06 ± 0.36	
		C : 22						IL-6	IL-6 (ng/L) : before: 4.84 ± 3.67 after: 3.45 ± 1.69	IL-6 (ng/L) : before: 4.38 ± 2.67; after: 4.58 ± 1.85	

Farajbaksh et al. [31]	Iran	M : 16	30–70 yrs	Parallel	Sesame oil (30 mL/d)	Placebo (sunflower oil)	8	hs-CRP	hs-CRP (mg/L) : before: 43.8 ± 13.4; after: 39.6 ± 17	Hs-CRP (mg/L) : before: 43.5 ± 17.4; after: 43.9 ± 20.1	47 patients with metabolic syndrome
		F : 31	S : 48.04 ± 7.67								
		S : 24 (7/17)	C : 50.17 ± 7.6								
		C : 23 (9/14)									

Da silva barbosa et al. [30]	Brazil	M : 20	16–18 yrs		Sesame seed (40 g/d)	Placebo (honey, maltodextrin, cow milk, and artificial caramel food coloring)	4	hs-CRP	hs-CRP (mg/L) : before: 3.66 ± 1.4; after: 0.8 ± 1.0	hs-CRP (mg/L) : before: 3.31 ± 1.8; after: 0.6 ± 0.4	20 semiprofessional soccer players
		S : 10									
		C : 10									

Mohammad Shahi et al. [35]	Iran	F + M: 48	30–60 yrs	Parallel	Sesamin capsule (200 mg/d)	Placebo capsules (starch)	8	hs-CRP	hs-crp (mg/L) : before: 2.83 ± 1.35; after: 2.53 ± 1.44	Hs-CRP (mg/L) : before: 2.80 ± 1.25; after: 2.92 ± 1.3	44 type 2 diabetes mellitus
		S : 24	S : 50 ± 12.3					TNF-*α*	TNF (ng/L) before: 1.92 ± 0.76; after: 1.3 ± 0.27	TNF (ng/L) : before: 1.66 ± 0.76; after: 1.86 ± 0.9	
		C : 24	C : 51.72 ± 12.24					IL-6	IL-6 (ng/L) : before: 20.19 ± 12.1; after: 17.2 ± 9.13	IL-6 (ng/L) : before: 21.26 ± 10.73 after: 22.19 ± 11.14	
								IL-6	Adiponectin (*µ*g/mL) : before: 6.21 ± 1.33; after: 7.34 ± 2.88	Adiponectin (*µ*g/L) : before: 6.60 ± 1.62; after: 6.19 ± 1.10	

Haghighian et al. [32]	Iran	F + M: 45	50–70 yrs	Parallel	Sesame seed (40 g/d) + acetaminophen	Placebo (glucosamine) + acetaminophen	8	hs-CRP	hs-CRP (mg/L) : before: 1.45 ± 1.12; after: 1.42 ± 1.32	hs-CRP (mg/L) : before: 1.64 ± 1.19; after: 1.68 ± 0.87	45 patients with knee osteoarthritis
		S : 22	S : 56.90 ± 6.39					IL-6	IL-6 (ng/L) : before: 2.29 ± 0.82; after: 0.38 ± 0.05	IL-6 (ng/L) : before: 2.43 ± 0.68; after: 1.53 ± 0.04	
		C : 23	C : 58.27 ± 7.84								

Karatzi et al. [34]	Greece	M : 30	S : 49.8 ± 8.46	Parallel	Sesame oil (35 g/d)	Control	8	CRP	CRP (mg/L) : before: 1.8 ± 1.8; after: 2.0 ± 2.3	CRP (mg/L) : before: 1.9 ± 2.3; after: 2.0 ± 2.5	30 hypertension men
		S : 17	C : 56.8 ± 12					TNF-*α*	TNF (ng/L) : before: 0.77 ± 1.0; after: 0.96 ± 1.5	TNF (ng/L) : before: 0.31 ± 0.51; after: 0.54 ± 0.61	
		C : 13									

Wu et al. [24]	Australia	F + M: 3	S: 54.7 ± 8.6	Crossover	Sesame seed (25 g/d)	Placebo	5	hs-CRP	hs-CRP (mg/L) : before: 2.1 ± 2.5; after: 1.89 ± 2.06	hs-CRP (mg/L) : before: 1.98 ± 2.29; after: 2.02 ± 2.42	38 overweight men and women
		S : 38	C : 54.7 ± 8.6					TNF-*α*	TNF-*α* (ng/L) : before: 1.85 ± 0.59; after: 1.83 ± 0.58	TNF-*α* (ng/L) : before: 1.83 ± 0.61; after: 1.8 ± 0.59	
		C : 38						IL-6	IL-6 (ng/L) : before: 2.08 ± 1.16; after: 2.05 ± 1.03	IL-6 (ng/L) : before: 2.14 ± 1.3; after: 2.25 ± 1.33	

^1^Values are overall ranges and means ± SDs in each group. ^2^Values are means ± SDs. C: control; CRP: C-reactive protein; F: female; hs-CRP: highly sensitive C-reactive protein; IL-6: interleukin-6; M: male; S: sesame; and TNF-*α*: tumor necrosis factor-*α*.

**Table 2:** Cochrane's risk of bias assessment for randomized controlled trials on the effect of sesame consumption on inflammatory biomarkers in human^1^.

Reference	Random sequence generation	Allocation concealment	Blinding of participants, personnel, and outcome assessors	Incomplete outcome data	Selective outcome reporting	Other sources of bias
Helli et al. [33]	L	L	L	L	L	L
Farajbakhsh et al. [31]	L	H	L	L	L	L
Da Silva Barbosa et al. [30]	L	H	L	L	L	L
Mohammad Shahi al. [35]	L	H	L	L	L	L
Haghighian et al. [32]	L	H	H	L	L	L
Karatzi et al. [34]	L	H	H	L	L	L
Wu et al. [24]	L	L	L	L	L	L

^1^H: high risk of bias; L: low risk of bias; and U: unclear risk of bias.

**Table 3:** Pooled estimates of the effects of sesame consumption on inflammatory biomarkers within different subgroups^3^.

	Number of trials			WMD (95% CI)			*P* value			*P*-heterogeneity			*I* ^2^ (%)		
	CRP	TNF	IL-6	CRP	TNF	IL-6	CRP	TNF	IL-6	CRP	TNF	IL-6	CRP	TNF	IL-6
Total	7	4	4	−0.55 (−1.22, 0.12)	−0.34 (−0.77, 0.08)	−0.90 (−1.71, −0.09)	0.109	0.111	0.029	<0.001	<0.001	0.002	75.1	88.4	80.4

Baseline serum biomarker¹															
<10 mg/L	5	—	2	−0.20 (−0.48, 0.06)	—	−0.56 (−1.41, 0.28)	0.138	—	0.193	0.831	—	0.158	0	—	49.8
≥10 mg/L	2	—	2	−5.91 (−8.25, −3.58)	—	−1.94 (−3.57, −0.30)	<0.001	—	0.020	0.666	—	0.029	0	—	78.9

Baseline BMI															
≥30 kg/m^2^	3	2	1	−3.40 (−8.16, 1.35)	−0.24 (−0.77, 0.28)	−0.73 (−2.13, 0.66)	0.161	0.363	0.303	<0.001	0.002	—	90.5	89.5	—
<30 kg/m^2^	4	2	3	−0.19 (−0.49, 0.10)	−0.45 (−1.21, 0.31)	−1.75 (−4.23, 0.73)	0.193	0.245	0.167	0.693	0.008	0.266	0	85.9	24.5

Type of intervention															
Sesame capsule	2	2	2	−0.62 (−1.09, −0.15)	−0.67 (−0.95, −0.38)	−1.94 (−3.57, −0.30)	0.271	<0.001	0.020	<0.001	0.190	0.020	94.8	41.9	14.9
Sesame seed	3	1	2	−0.12 (−0.47, 0.22)	0.01 (−0.15, 0.17)	−0.56 (−1.41, 0.28)	0.476	0.907	0.193	0.908	—	0.193	0	—	89.8
Sesame oil	2	1	—	−0.02 (−1.07, 1.02)	−0.04 (−0.52, 0.44)	—	0.582	0.871	—	0.157	—	—	50.1	—	—

Duration of treatment															
<8 wk	3	2	2	−1.73 (−3.92, 0.45)	−0.24 (−0.77, 0.28)	−0.73 (−2.13, 0.66)	0.120	0.363	0.303	<0.001	0.002	0.029	90.1	89.5	78.9
=8 wk	4	2	2	−0.21 (−0.55, 0.12)	−0.45 (−1.21, 0.31)	−1.75 (−4.23, 0.73)	0.216	0.245	0.167	0.356	0.008	0.158	7.5	85.9	49.8

Geographic region															
Asia	4	2	3	−1.38 (−2.70, −0.06)	−0.67 (−0.95, −0.38)	−1.23 (−1.95, −0.52)	0.039	<0.001	0.001	<0.001	0.190	0.266	87.3	41.9	24.5
Non-Asia	3	2	1	−0.14 (−0.65, 0.35)	0.005 (−0.15, 0.16)	−0.14 (−0.48, 0.20)	0.559	0.955	0.428	0.861	0.848	—	0	0	—

Sample size															
<40	3	2	1	−0.14 (−0.65, 0.35)	0.005 (−0.15, 0.16)	−0.14 (−0.48, 0.20)	0.559	0.955	0.428	0.861	0.848	0.428	0	0	—
≥40	4	2	3	−1.38 (−2.70, −0.06)	−0.67 (−0.95, −0.38)	−1.23 (−1.95, −0.52)	0.039	<0.001	<0.001	<0.001	0.190	0.001	87.3	41.9	24.5

Sex															
Men	2	1	−	−0.02 (−0.77, 0.72)	−0.04 (−0.52, 0.44)	—	0.950	0.871	—	—	—	—	0	—	—
Women	1	1	1	-6.12 (−8.63, −3.61)	−0.53 (−0.82, −0.23)	−1.59 (−2.84, −0.33)	<0.001	0.001	0.013	0.411	—	—	—	—	—
Both	4	2	3	−0.24 (−0.53, 0.04)	−0.39 (−1.20, 0.41)	−0.72 (−1.62, 0.17)	0.103	0.342	0.116	0.745	<0.001	0.002	0	95.2	84.1

^1^<10 mg/L, ≥10 mg/L for CRP, <2 ng/L, ≥2 ng/L for TNF, <3 ng/L, ≥3 ng/L for IL-6, ^2^BMI: body mass index; CI: confidence interval; CRP: C-reactive protein; IL-6: interleukin-6; TNF-*α*: tumor necrosis factor-*α*; and WMD: weighted mean difference.

## Data Availability

Data will be made available if deemed necessary upon request to the corresponding author.

## Supplementary Materials

Supplementary Materials
*Supplementary Figure 1*. Analysis of the influence of sesame consumption on serum CRP concentrations in humans. CI: confidence interval; CRP: C-reactive protein. *Supplementary Figure 2*. Analysis of the influence of sesame consumption on serum IL-6 concentrations in humans. CI: confidence interval; IL-6: interleukin-6. *Supplementary Figure 3*. Analysis of the influence of sesame consumption on serum TNF concentrations in humans. CI: confidence interval; TNF: tumor necrosis factor. *Supplementary Figure 4*. Funnel plot for assessing publication bias in the studies reporting the effects of sesame consumption on serum CRP concentrations in humans. CRP: C-reactive protein; SE: standard error; WMD: weighted mean difference. *Supplementary Figure 5*. Funnel plot for assessing publication bias in the studies reporting the effects of sesame consumption on serum IL-6 concentrations in humans. IL-6: interleukin-6; SE: standard error; and WMD: weighted mean difference. *Supplementary Figure 6*. Funnel plot for assessing publication bias in the studies reporting the effects of sesame consumption on serum TNF concentrations in humans. TNF: tumor necrosis factor; SE: standard error; and WMD: weighted mean difference.
