# Supplementary material for: Effects of Sesame Consumption on Inflammatory Biomarkers in Humans: A Systematic Review and Meta-Analysis of Randomized Controlled Trials
Source: Evid Based Complement Alternat Med. 2021 Nov 1;2021:6622981. doi: 10.1155/2021/6622981 (PMC8575626; doi:10.1155/2021/6622981)
Supplement: Supplementary Materials — Supplementary Figure 1. Analysis of the influence of sesame consumption on serum CRP concentrations in humans. CI: confidence interval; CRP: C-reactive protein. Supplementary Figure 2. Analysis of the influence of sesame consumption on serum IL-6 concentrations in humans. CI: confidence interval; IL-6: interleukin-6. Supplementary Figure 3. Analysis of the influence of sesame consumption on serum TNF concentrations in humans. CI: confidence interval; TNF: tumor necrosis factor. Supplementary Figure 4. Funnel plot for assessing publication bias in the studies reporting the effects of sesame consumption on serum CRP concentrations in humans. CRP: C-reactive protein; SE: standard error; WMD: weighted mean difference. Supplementary Figure 5. Funnel plot for assessing publication bias in the studies reporting the effects of sesame consumption on serum IL-6 concentrations in humans. IL-6: interleukin-6; SE: standard error; and WMD: weighted mean difference. Supplementary Figure 6. Funnel plot for assessing publication bias in the studies reporting the effects of sesame consumption on serum TNF concentrations in humans. TNF: tumor necrosis factor; SE: standard error; and WMD: weighted mean difference. [file 6622981.f1.docx]

**Supplementary Figure 1.** Analysis of the influence of sesame consumption on serum CRP concentrations in humans. CI, confidence interval; CRP, C-reactive protein

**Supplementary Figure 2.** Analysis of the influence of sesame consumption on serum IL-6 concentrations in humans. CI, confidence interval; IL-6, interleukin-6

**Supplementary Figure 3.** Analysis of the influence of sesame consumption on serum TNF concentrations in humans. CI, confidence interval; TNF, tumor necrosis factor


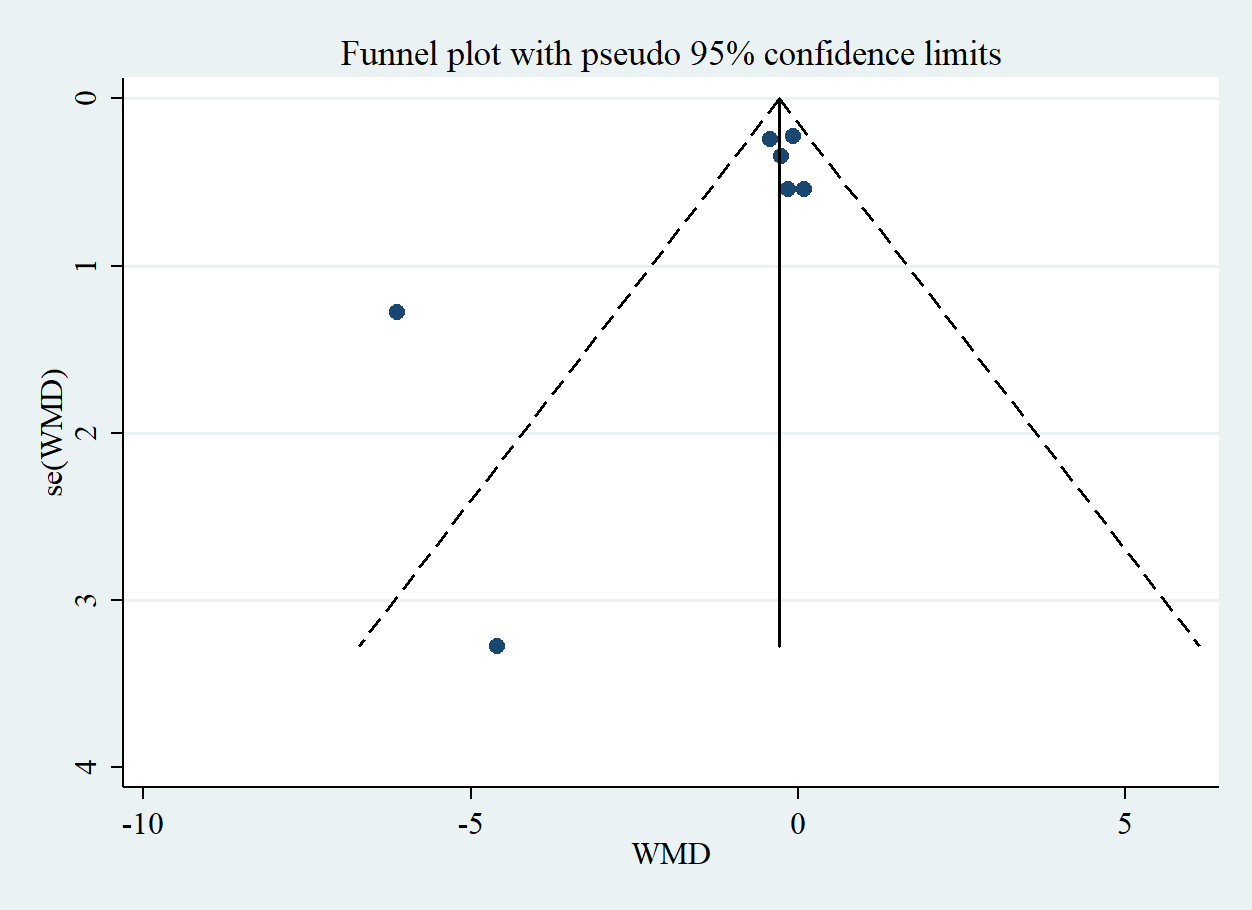


**Supplementary Figure 4.** Funnel plot for assessing publication bias in the studies reporting the effects of sesame consumption on serum CRP concentrations in humans. CRP, C-reactive protein; SE, standard error; WMD, weighted mean difference

**
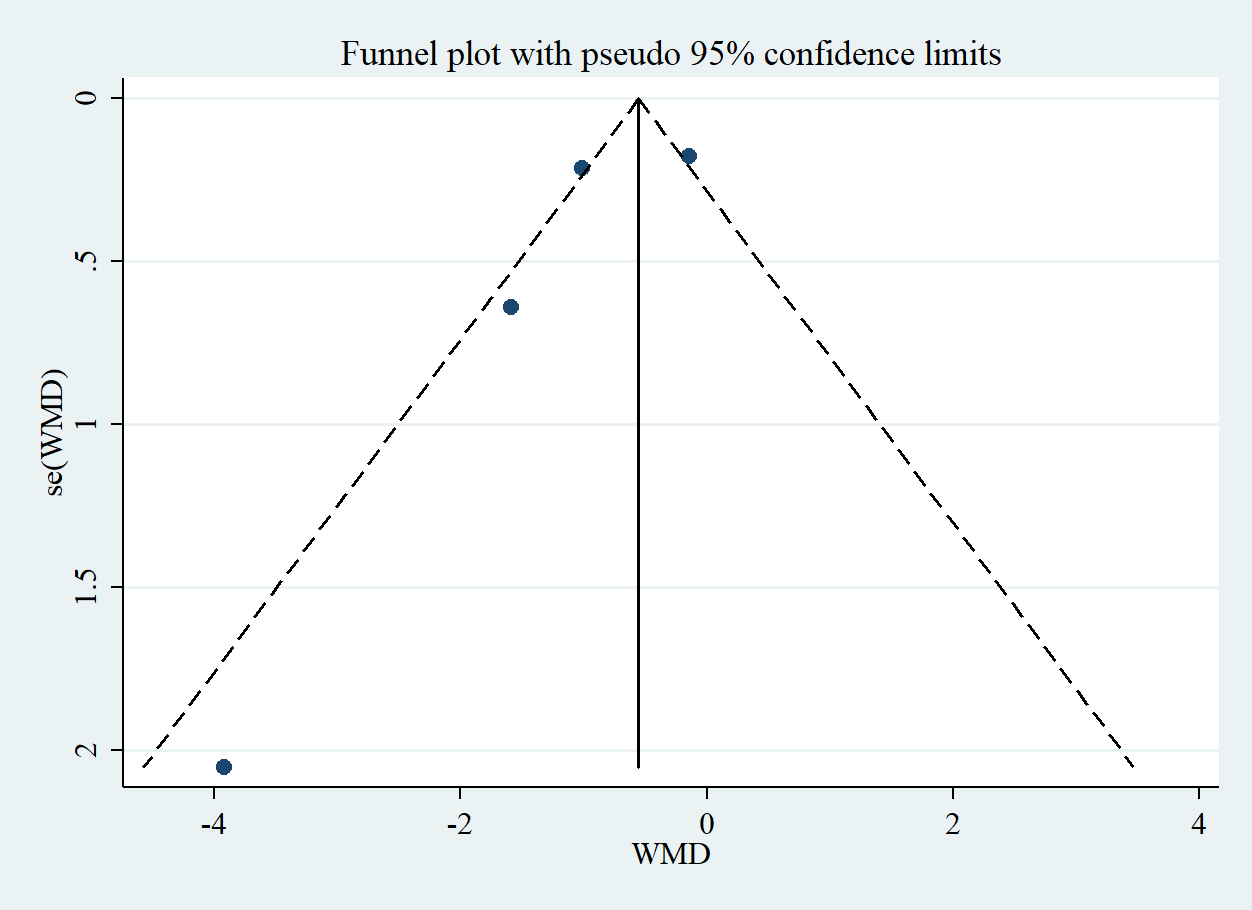
**

**Supplementary Figure 5.** Funnel plot for assessing publication bias in the studies reporting the effects of sesame consumption on serum IL-6 concentrations in humans IL-6, interleukin-6 SE, standard error; WMD, weighted mean difference


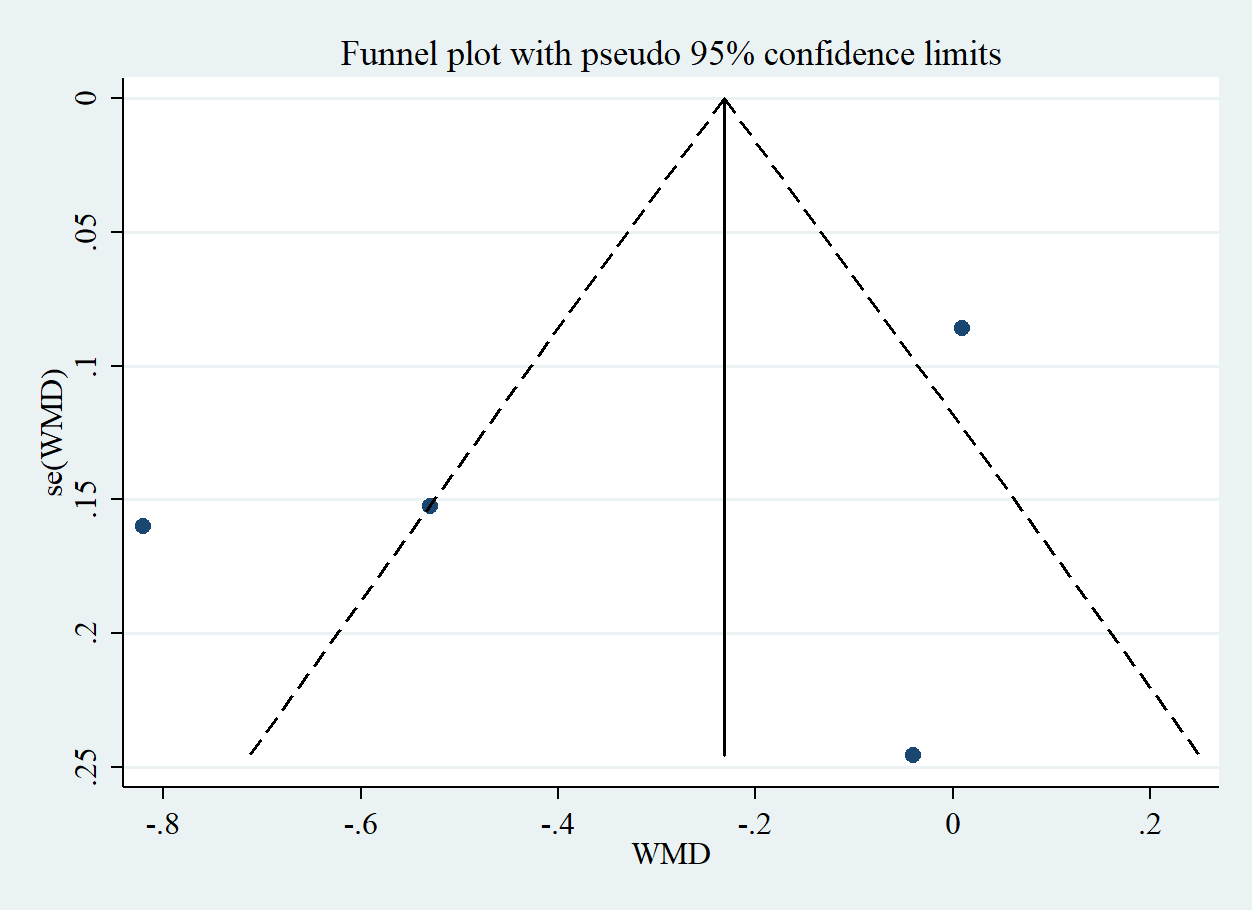


**Supplementary Figure 6.** Funnel plot for assessing publication bias in the studies reporting the effects of sesame consumption on serum TNF concentrations in humans. TNF, tumor necrosis factor SE, standard error; WMD, weighted mean difference

;;;
